# Exploring the mental health impact of COVID-19 on parents of young children: anxiety, depression, and contributing factors

**DOI:** 10.1186/s12889-025-23467-9

**Published:** 2025-06-26

**Authors:** Elisa Rodríguez-Toscano, Daniel Schleicher, Angelika Ecker, Stephanie Kandsperger, Romuald Brunner, Irina Jarvers

**Affiliations:** 1 Department of Experimental Psychology, Cognitive Processes and Speech Therapy, Universidad Complutense de Madrid (UCM), Madrid, Spain; 2 Department of Child and Adolescent Psychiatry, Institute of Psychiatry and Mental Health, Hospital General Universitario Gregorio Marañón School of Medicine, Universidad Complutense, IiSGM, CIBERSAM, Madrid, Spain; 3 Department of Child and Adolescent Psychiatry and Psychotherapy, University of Regensburg, Universitaetsstr. 84, Regensburg, 93053 Germany

**Keywords:** COVID-19 pandemic, Parental mental health, Anxiety, Depression, Pandemic-related stressors, Parents of preschool children

## Abstract

**Background:**

The COVID-19 pandemic led to a global shutdown, with most countries implementing widespread lockdowns. While such measures were essential in curbing the spread of COVID-19, their impact on the mental health of parents with preschool-aged children is not yet sufficiently understood. This study aimed to assess anxiety and depression symptoms in parents during and after the COVID-19 lockdowns, and to examine how demographic and pandemic-related factors influenced these outcomes.

**Method:**

A sample of 128 parents in Germany with preschool children (aged 2 to 6 years) participated in an online survey. The survey assessed three key periods: before the nationwide lockdowns (retrospectively), during the most challenging phase of the lockdown (retrospectively), and after the lockdowns were lifted. Symptoms of depression and anxiety were investigated with validated questionnaires and pandemic-specific stressors (e.g., isolation of child) with a self-developed set of 23 items. Descriptive statistics, group comparisons, and hierarchical multiple regression analyses were conducted.

**Results:**

Symptoms of anxiety and depression significantly increased during lockdowns in comparison to before pandemic onset and remained elevated after restrictions eased. Key predictors of poorer mental health included pre-pandemic symptoms, lower parental education, concerns about access to primary care, and children's social isolation during lockdowns. Demographic factors alone were not consistently significant predictors.

**Conclusions:**

Symptoms of anxiety and depression significantly increased during lockdowns compared to before pandemic onset and remained elevated after restrictions eased. COVID-19-related stressors played a greater role in parental mental health outcomes than demographic variables. These findings emphasize the need for early identification of at-risk families and the development of targeted interventions to support parental well-being in future public health emergencies.

## Introduction

In March 2020, the World Health Organization officially recognized the spread of COVID-19 as a global pandemic [1]. Consequently, various governments implemented public health measures and restrictions to curb the transmission of SARS-CoV-2. These measures involved travel bans, social distancing, and the closure of workplaces, daycare centres, schools, preschools, and universities. Specifically in Germany, two nationwide lockdowns were enforced: the first from March to May 2020, and the second from December 2020 to May 2021. Both lockdowns involved restrictions on social interactions, including limitations on gatherings, physical distancing measures, and stay-at-home orders, which reduced opportunities for face-to-face contact with friends, extended family, and colleagues. Additionally, it involved closure of shops, restaurants, schools, and preschools, which may have resulted in disrupted daily routines, limited access to support networks, and increased social isolation.

The pandemic and the accompanying restrictions have been shown to result in decreased general well-being and an increase in affective and stress-related symptoms, including depression, generalized anxiety disorder or insomnia [2, 3]. These effects have been especially pronounced in specific subgroups, including parents [4]. Research indicates that parents experienced more burnout symptoms and more mental health problems than non-parents [5, 6]. Studies have consistently reported elevated levels of parental stress [7, 8] and clinically significant depressive and anxious symptomatology linked to the COVID-19 pandemic [9–13]. This elevation of anxious and depressive symptomatology is maintained even during phases of the pandemic with fewer or no restrictions [14] and also after the COVID-19 lockdowns [15, 16]. A recent review highlighted that the prevalence rates of clinically elevated depression and anxiety symptoms among mothers of young children (under age 5) during the pandemic were 26.9% and 41.9%, respectively [17]. These figures indicate an increase compared to pre-pandemic estimates, which reported that approximately 15% of parents experienced symptoms of depression or anxiety [18].

In this context, studies have aimed to delineate which demographic and COVID-related factors most significantly influence parental mental health. Findings suggest that job -related variables such as reduced income during the pandemic [19, 20], income insecurity [7], and experiencing job loss [21] have notable adverse effects on mental health during this period. Moreover, variables intrinsic to the parents themselves, including parents’ history of mental illness [11], are related to higher levels of anxiety and depression symptomatology in the context of COVID-19 lockdowns. Anxious symptomatology is more prevalent among mothers and older individuals [17, 19], while depression levels are higher among those from racially minoritized groups [17]. Interestingly, higher education levels also seem to influence the development of mental health symptoms during the pandemic, with more educated parents experiencing lower levels of distress [17, 21]. This may be partly due to the fact that parents with higher education levels are more likely to have jobs that allow remote work, which can create unique stressors [21].

Additionally, some child-related factors have been shown to influence parents’ mental health. Parents and caregivers who had to homeschool their children during the COVID-19 pandemic lockdowns reported significantly higher levels of psychological distress and work/social impairment compared to those who did not homeschool or had no school-aged children [22]. Within this framework, parents of younger children and those at earlier stages of their child’s education experienced greater challenges during pandemic than parents of older children [11].

Even after the most challenging phases of the pandemic were over and pandemic protocols were lifted, parents have continued to experience similar levels of anxiety and depression [15], which are closely linked to ongoing parental stress [23]. However, literature examining risk factors affecting parental mental health post-pandemic is limited. Buechel et al. [15] found that among mothers of toddlers, depression and anxious symptomatology at follow-up were predicted by mental health levels at baseline, family conflicts and fear of COVID-infection. Additionally, Skripkauskaite et al. [24] identified different trajectories of demographic and clinical variables during and after the pandemic. Specifically, while anxiety symptoms increased among parents of 4–10-year-olds between April 2020 and January 2021, they decreased among parents of children under 4 years old. Conversely, depression levels rose among parents of children aged 11–17 years compared to those with 4–10-year-olds.

Despite prior work examining the mental health of preschool children’s parents in light of the pandemic, most of these studies only reported on the first pandemic year [6, 13, 25] whereas longitudinal studies [7, 22] mainly compared their results to pre-pandemic surveys and do not examine which factors contribute to the maintaining of anxiety and depressive symptoms after the discontinuation of the pandemic protocols.

The purpose of the current study was to examine whether parents of preschool children reported significantly higher levels of anxiety and depressive symptoms during the most critical phase of the second COVID-19 lockdown in Germany, and whether these symptoms persisted two weeks after the lockdown had ended. Additionally, the study sought to examine how a selected set of demographic and COVID-related variables may influence these mental health outcomes. It was hypothesized that anxiety and depression levels would be elevated during and shortly after the lockdown compared to pre-pandemic levels. While numerous contributing factors have been identified in the literature, our analysis focused on a core group of variables that were most relevant to our study population and feasible to measure within our design. These included parental education, child age, and specific experiences related to the lockdown period.

## Methods

### Participants

For the present study, participants were recruited considering the following inclusion criteria a) being the parent of a child between 2 and 6 years of age; and b) attendance of formal early education before the onset of the COVID-19 pandemic lockdown. While attending formal early education was not central to our research question, it served to define a more homogeneous sample in terms of pre-pandemic routines. Exclusion criterion was not living in Germany. The age range of 2–6 years was chosen to include children in both nursery (German: Krippe, ages 0–3) who may already show early behavioural difficulties, and children in kindergarten (ages 3–6) to cover the whole formal early education range.

Participating parents were recruited via local preschools, flyers at supermarkets, notices on social media platforms and parent groups. Regarding local preschools, educators received flyers that were printed out and distributed among parents or sent digitally over parent mailing lists including a link to the survey and a QR code.

A total of 120 preschools catering to children aged 0–6 years were contacted, including 53 preschools for children aged 0–3 years and 67 preschools for children aged 3–6 years. Of these, 69 preschools (57.5%) agreed to participate. In total, 128 parents (112 mothers, 87.5%) were enrolled into the study in the final sample. An additional 16 parents consented to participate but did not provide any data. Among the 128 data sets collected, 113 were complete, including all items and questionnaires (88.3%). The sample size was based on an a priori power analysis conducted for a previously published study using the same dataset, focusing on the impact of preschool closures on children's mental health [26]. All participants gave their informed consent for participation and completed the questionnaires electronically. Email addresses were voluntarily entered into a separate survey for a raffle and could not be matched to answers within the main survey. Data was collected anonymously without IP addresses or GPS tracking. The study was approved by the Ethics Committee of the University of Regensburg (20–1916-101) and preregistered in the German Clinical Trials Register (DRKS; DRKS00023812).

### Measures

The present research was carried out through an online survey using the PsyToolKit software (www.psytoolkit.org/) [27, 28]. The survey lasted about 45 min, and upon completion, parents could provide a valid email address to enter into a raffle for a chance to win one of 20 vouchers, each worth 25 euros.

The measures used in the present study included demographic variables, COVID-related variables and parents’ mental health traits, including parental stress, parental anxious symptomatology and parental depressive symptomatology.

Parents were asked about three distinct time periods: two weeks before the second nationwide lockdown (T1), two weeks during the peak of that lockdown (T2), and the most recent two weeks following the lockdown (T3). T1 and T2 were evaluated retrospectively.

Data was collected between June 2021 and February 2022 after the second nationwide lockdown in Germany.

#### Demographic variables

Demographic variables were assessed including the following information a) *general information* during the lockdown (sex of the respondent, parents’ age, child’s age, child’s sex, whether the child has siblings and the number of people living in the household); and b) *respondents’ characteristics* (relationship status, parental education, employment, whether they worked in a healthcare institution, and their financial situation).

#### COVID-related variables

COVID-related variables were based on self-developed items from a previous publication on parental mental health during COVID-19 by Ecker et al. [29]. The following COVID-related variables were examined:

- *Risk variables*: Whether the parent identified as part of a social/ethnic minority and whether they had tested positive for COVID-19.
- *Pandemic-induced changes*: Whether the parent experienced changes in their work hours during the COVID-19 pandemic, including working more or less than usual, being placed on short-time work, or working from home.
- *Personal worries:* Parents were asked to respond using the options "Not at all,""A little,""Quite a bit," or "Very much.", coded 1–4. The survey assessed whether the parents were afraid of contracting COVID-19, worried about the impact of COVID-19 on primary care availability, felt they had received sufficient information about COVID-19 and resulting changes, recognized the possibility that the lockdown could last longer than expected, and whether the government measures were correct and necessary.
- *Child-related worries:* Parents rated their concern about their child’s risk of COVID-19 infection on a five-point Likert scale (1 ="Do not agree at all" to 5 = "Totally agree"). Items assessed whether parents were worried about their child becoming infected, their own ability to care for the child if infected, the child’s understanding of government measures, the child’s own worries about infection, concern for the parent’s or others’ well-being, and distress caused by the general mask mandate.
-*Children’s activities during lockdown:* On a four-point scale (1 = “Not at all” to 4 = “Very much”), parents reported how often their child spent time alone, went outdoors, did sports, saw friends, had contact with other households, and watched TV during the most recent lockdown.

#### Parental mental health

Parental mental health was assessed through a measure for anxious symptomatology using the Generalized Anxiety Disorder 7 (GAD-7) [30], depressive symptomatology through the Center for Epidemiologic Studies Depression Scale Revised (CESD-R) [31], revised by Eaton and his collaborators [32], and for parental stress the Parental Stress Scale (PSS) [33].

- *Parental anxious symptomatology*. The GAD-7 is a self-report questionnaire consisting of seven items. Scores range from 0 to 21, and based on established clinical cutoffs [30] 0–4 indicate low anxiety, 5–9 represent mild anxiety, 10–14 reflect moderate anxiety, and 15–21 denote high anxiety levels. It is frequently utilized to assess generalized anxiety disorder and has demonstrated strong reliability in evaluating general anxiety symptoms in both clinical [34] and community samples [35], exhibiting high internal consistency across all groups (Cronbach’s α = 0.89) [35]. In this study, parental anxious symptomatology was assessed at the time points T1, T2 and T3.
- *Parental depressive symptomatology*. The CESD-R is a comprehensive questionnaire designed to assess depression across nine different domains: Sadness (Dysphoria), Loss of Interest (Anhedonia), Appetite, Sleep, Thinking/Concentration, Guilt (Worthlessness), Tiredness (Fatigue), Movement (Agitation) and Suicidal Ideation. It includes 20 self-report items, with scores ranging from 0 to 60. Scores below 16 suggest no clinical significance, whereas scores above 16 indicate subthreshold depression based on established clinical cutoffs [36]. In the present study, the CESD-R was used as a dimensional measure of symptomatology. The CESD-R has demonstrated good internal consistency with a Cronbach’s alpha of 0.82 [37]. In this study, parental depressive symptomatology was assessed at the time points T1, T2 and T3.
- *Parental stress*. The PSS is a questionnaire that evaluates stress experienced by parents in their role. It comprises 18 items that address both the positive aspects of parenthood (i.e., I feel close to my children) and the negative aspects (i.e., caring for my child(ren) sometimes takes more time and energy than I have to give) [33]. The PSS has demonstrated good internal consistency for both the original English [33, 38–40] and the German translation [41]. In this study, parental stress was assessed at the time points T1 and T2.

### Statistical analysis

For the descriptive analysis of the demographic and clinical variables, we employed descriptive statistics (mean, standard deviation, and proportion, when appropriate). Clinical values in the three time points were compared using Analysis of Variances (ANOVAs) and post hoc pairwise comparisons with applied Bonferroni correction according to Levene’s test.

The relationship between anxious/depressive symptomatology and the demographic and COVID-related variables was explored using Mann Whitney *U* tests (2 categories) and Kruskal–Wallis *H* tests (3 or more categories) for categorical variables and Spearman correlations for quantitative variables. The normality of variables was explored through the Kolmogorov–Smirnov test.

To assess which factors contributed to an increase in anxious/depressive symptomatology during the most critical phase of the second nationwide lockdown (T2) and after the second lockdown (T3) we employed backwards-stepwise linear regressions to systematically simplify the model while retaining key predictors. We assessed multicollinearity using Variance Inflation Factors (VIF < 5) and checked linear regression assumptions by examining residual normality (Q-Q plots, histograms), homoscedasticity (residuals vs. fitted values), and linearity (partial regression plots). Four separate multiple regression models were run for each mental health score used as a dependent variable (i.e. anxious symptomatology at T2, anxious symptomatology at T3, depressive symptomatology at T2, depressive symptomatology at T3). To determine the best set of demographic and COVID-related variables that would explain the maximum variance for anxious and depressive symptomatology in the linear regression models, we included only those demographic and COVID-related variables that were significantly associated with mental health variables in the previous analyses. Additionally, in the regression models, we controlled for parental stress measures evaluated at T1 and anxious/depressive symptomatology evaluated at T1 (anxious T1 for anxious symptomatology models and depressive T1 for depressive symptomatology models).

Multiple comparisons were corrected for using the Bonferroni correction and the significance threshold was set at *p* ≤ 0.05 bilaterally. Effect sizes were interpreted using Cohen’s d, with values of 0.2, 0.5, and 0.8 representing small, medium, and large effects, respectively [42]. Statistical analyses were conducted using SPSS 27 (IBM Corp Released, 2021).

## Results

### Sample demographic and COVD-related characteristics

The majority of participants responding to the questionnaire were mothers (87.50%) with a mean age of 36.78 (*SD* = 4.75) years. Children displayed a mean age of 4.17 (*SD* = 1.12) years and 38.30% were female. Within the sample, over 54.30% possessed at least a bachelor’s degree and most of the respondents lived with someone (91.40%) and were working (81.20%) at the time of the survey.

Concerning the COVID variables, 41.40% of the parents were working in home office due to the COVID-19 pandemic and 25.80% had to work more and 11.70% less than usual.

Participants'main personal concerns centred on receiving sufficient information about COVID-19 and resulting changes, as well as the possibility of an extended lockdown. More than 80% of participants rated these worries as “quite a bit” or “very much”. When it came to concerns related to their children, the main worries included the risk of either the child or the parent becoming infected by COVID-19, the inability to care for the child if the parent fell ill, and whether the child fully understood the government’s measures. Approximately 50% of parents expressed the highest level of concern for these issues.

Regarding children’s activities during lockdown, parents reported that the most prevalent activities were spending time outdoors and watching TV. Conversely, the least frequent activities were seeing friends and having contact with people from other households. A demographic and COVID-related overview of the sample is depicted in Table 1.

**Table 1:** Description of demographic, COVID related and clinical variables of the sample

**Sociodemographic variables**	
*** General information***	***n*** ** (%)/** ***M (SD)***
*** Respondant (mother)***	112 (87.5)
Age (parent)	36.78 (4.75)
Age (child)	4.17 (1.12)
Sex (child, female)	49 (38.3)
Siblings	99 (77.3)
Household size	3.94 (0.84)
*** Respondants’ characteristics***	***n*** ** (%)**
*** Relationship***	
* Single*	6 (4.7)
* Living with somebody*	117 (91.4)
* Separated*	5 (3.9)
Education	
* 9 years school education*	5 (3.9)
* 10 years school education*	8 (6.3)
* Vocational school*	16 (12.5)
* A-levels*	34 (26.6)
* University*	65 (50.8)
Currently working	104 (81.2)
Job in healthcare	29 (22.7)
Income	
* less*	11 (8.6)
* same*	107 (83.6)
* more*	10 (7.8)
**Covid-related variables**	
*** Risk variables***	***n*** ** (%)**
I consider myself part of a social/ethnic minority	12 (9.4)
I tested COVID-19 positive	19 (14.8)
*** Changes due to covid***	***n*** ** (%)**
I had to work more than usual	33 (25.8)
I was on short-time work	14 (10.9)
I was working in home office	53 (41.4)
I had to work less than usual	15 (11.7)
***Worries related to themselves***	***M (SD)***
I am afraid of getting infected	2.30 (0.77)
I am worried that primary care is endangered	1.84 (0.85)
I have received enough information and resulting changes	3.30 (0.76)
I was aware that the lockdown may be longer than anticipated	3.23 (0.84)
I consider the measures taken by the government correct and necessary	3.08 (0.87)
***Worries related to child***	***M (SD)***
I am worried that my child may get infected	2.72 (0.90)
I am worried that I may get infected and won't be able to take care of my child	2.48 (0.90)
My child has understood the government's measures^a^	3.05 (1.42)
My child is worried about getting infected^a^	1.98 (1.26)
My child is worried about me or about his friends/acquaintances^a^	2.17 (1.35)
My child is unsettled by the general mask mandate^a^	1.88 (1.15)
***Children activities during Lockdown***	***M (SD)***
My child was alone	1.84 (0.95)
My child was outdoors	3.28 (0.80)
My child did sports	2.40 (0.86)
My child was able to see friends	1.95 (0.68)
My child was in contact with people from other households	2.15 (0.65)
My child has watched TV	2.82 (0.84)
**Clinical variables**	***M (SD)***
PSS	
* T1*	36.92 (8.77)
* T2*	43.41 (12.10)
GAD-7	
* T1*	3.92 (3.86)
* T2*	7.1 (5.21)
* T3*	5.3 (4.75)
CESD-R	
* T1*	6.48 (8.80)
* T2*	16.94 (12.99)
* T3*	11.04 (11.70)

*CESD-R* Center for Epidemiologic Studies Depression Scale – Revised, *GAD-7* Generalized Anxiety Disorder 7, *PSS* Parental Stress Scale, *T1* two weeks before the second nationwide lockdown, *T2* two weeks during the peak of that lockdown, *T3* most recent two weeks following the lockdown

^a^Questions with answers ranging from 1 (do not agree at all) to 5 (totally agree) instead of 1 (not at all) to 4 (very much)

### Mental health symptomatology in the sample

**Fig. 1:**
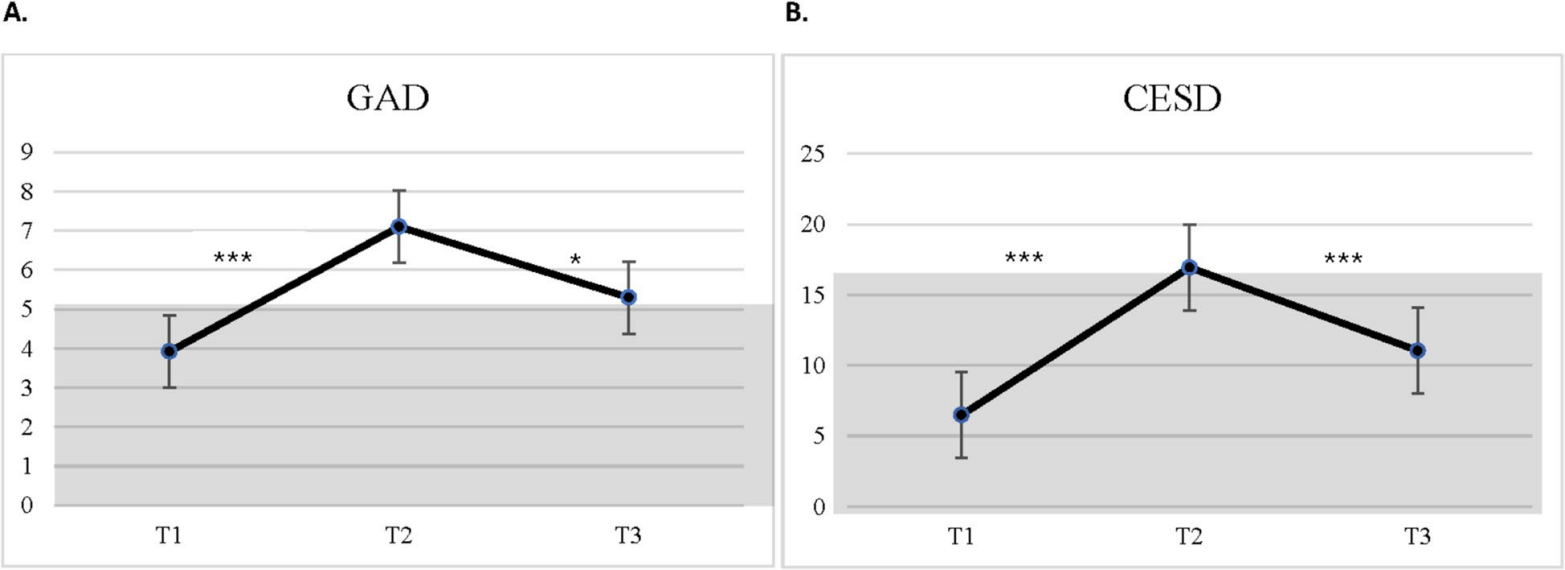
Overview of **A** depressive (CESD-R) and **B** anxious (GAD-7) symptomatology across T1 to T3 *Note.* *p < 0.05; **p < 0.01**; ***p <.001

In relation to anxious symptomatology, parents reported low levels at T1, which significantly increased during T2 and at T3 remained elevated in comparison to T1(*F*(2,338) = 13.32, *d′* = 0.70). However, scores at T3 significantly decreased compared to T2 and this difference was statistically significant, with a small-to-moderate effect size (Cohen’s *d* = 0.36). Depressive symptoms followed a similar trajectory, with a marked increase from T1 to T2 (*d′* = 0.94), surpassing the clinical threshold for depression, and a partial decrease by T3 (*F*(2,338) = 24.33, *d′* = 0.48). Despite the reduction, symptoms at T3 remained higher compared to T1 (*d′* = 0.44). A depiction of GAD-7 and CESD-R scores is provided in Fig. 1.

### Association between anxious and depressive symptomatology with demographic and COVID-related variables

Most demographic variables, such as parents’ or children’s age, were not significantly associated with anxious or depressive symptomatology (*p* > 0.05). One exception was education level: higher education was linked to lower depressive symptoms at T3. For complete statistics, see Table 2.

**Table 2:** Relationship between anxiety and depression with demographic and COVID-related variables

	**GAD (T2)**	**CESD-R (T2)**	**GAD (T3)**	**CESD-R (T3)**
**Demographic variables**				
*** House situation***				
Respondant (*U, p*)	468, 0.197	485, 0.259	550, 0.600	541.5, 0.547
Age (parent) (*r, p*)	0.03, 0.796	0.05, 0.623	0.06, 0.524	0.14, 0.141
Age (child) (*r, p*)	0.16, 0.093	0.11, 0.233	0.14, 0.135	0.08, 0.424
Sex (child) (*U, p*)	1133, 0.901	1138,0.933	1139, 0.935	1069, 0.551
Siblings (*r, p*)	−0.02, 0.828	0.01, 0.923	−0.15, 0.112	−0.04, 0.658
Household size (*r, p*)	0.07, 0.492	0.06, 0.558	−0.05, 0.603	0.00, 0.964
*** Respondants’ characteristics***				
Education (*r, p*)	−0.11, 0.234	−0.14, 0.150	−0.12, 0.341	−0.27, **0.032**
Relationship (K-W, *p*)	1.563, 0.458	4.229, 0.121	0.21, 0.901	1.79, 0.409
Currently working (K-W, *p*)	5.58, 0.061	6.61, **0.037**	0.05,0.977	0.67, 0.715
Job in healthcare (*U, p*)	1084.5, 0.751	997, 0.360	1121, 0.945	1069, 0.672
Income (K-W, *p*)	3.04, 0.219	2.96, 0.228	0.85, 0.655	1.18, 0.555
*** Risk variables***	***U, p***	***U, p***	***U, p***	***U, p***
I consider myself part of a social/ethnic minority	503, 0.573	479.5, 0.430	477.5, 0.417	497.5, 0.537
I tested COVID-19 positive	693, 0.722	687, 0.684	574, 0.171	583.5, 0.199
*** Changes due to COVID-19***	***U, p***	***U, p***	***U, p***	***U, p***
I had to work more than usual	1132, 0.571	1195, 0.882	948.5, 0.075	1069.5, 0.328
I was on short-time work	415.5, 0.158	417, 0.163	372, 0.066	412.5, 0.149
I was working in home office	1504.5, 0.881	1520,0.953	1509, 0.902	1488, 0.805
I had to work less than usual	379.55, **0.034**	366, **0.025**	492.5, 0.288	474, 0.217
*** Worries related to themselves***	***r, p***	***r, p***	***r, p***	***r, p***
I am afraid of getting infected	0.12, 0.200	−0.05, 0.623	0.19, 0.050	0.02, 0.850
I am worried that primary care is endangered	0.21,** 0.023**	0.13, 0.187	0.28,** 0.003**	0.13, 0.156
I have received enough information and resulting changes	0.02, 0.836	−0.05, 0.584	−0.07, 0.476	−0.20, **0.038**
I was aware that the lockdown may be longer than anticipated	−0.12, 0.194	−0.08, 0.415	−0.02, 0.839	−0.04, 0.711
I consider the measures taken by the government correct and necessary	−0.21, **0.027**	−0.26, **0.007**	−0.20, **0.033**	−0.20, **0.026**
*** Worries related to child***	***r, p***	***r, p***	***r, p***	***r, p***
I am worried that my child may get infected	0.12, 0195	0.02, 0.822	0.16, 0.086	0.07, 0.477
I am worried that I may get infected and won't be able to take care of my child	0.05, 0.598	−0.06, 0.504	0.16, 0.100	0.03, 0.746
I feel like my child has understood the government's measures	0.04, 0.695	0.02, 0.859	0.03, 0.741	−0.01, 0.955
My child is worried about getting infected	0.19, **0.044**	0.17, 0.065	0.15, 0.104	0.04, 0.643
My child is worried about me or about his/her friends/acquaintances	0.22,** 0.018**	0.20, **0.031**	0.20, **0.031**	0.10, 0.321
My child is unsettled by the general mask mandate	0.19, **0.040**	0.17, 0.066	0.17, 0.074	0.10, 0.385
*** Children activities during Lockdown***	***r, p***	***r, p***	***r, p***	***r, p***
My child was alone	0.30, **0.001**	0.25, **0.009**	0.33, < **0.001**	0.36, < **0.001**
My child was outdoors	−0.19, **0.045**	−0.12, 0.190	−0.19, **0.046**	−0.16, 0.097
My child did sports	−0.34, < **0.001**	−0.26,** 0.006**	−0.24, **0.010**	−0.27, **0.004**
My child was able to see friends	−0.14, 0.144	−0.18, 0.063	−0.14, 0.142	−0.19, **0.047**
My child was in contact with people from other households	−0.13, 0.162	−0.16, 0.101	−0.14, 0.134	−0.13, 0.165
My child has watched TV	0.09, 0.361	0.06, 0.545	0.15, 0.124	0.14, 0.138

The relationship between anxious/depressive symptomatology and the demographic and COVID-related variables was explored using Mann–Whitney U tests (2 categories) and Kruskal–Wallis H tests (3 or more categories) for categorical variables and Spearman correlations for quantitative variables

*CESD-R* Center for Epidemiologic Studies Depression Scale – Revised, *GAD-7* Generalized Anxiety Disorder 7, *T2* two weeks during the peak of that lockdown, *T3* most recent two weeks following the lockdown

Regarding COVID-related variables, the majority of risk and situational factors were not significantly associated with mental health symptoms. However, notable associations emerged: parents who worked less than usual during the pandemic exhibited higher anxiety and depression symptoms at T2. Self-related worries—such as concern about access to primary care and distrust in government measures—were consistently linked to elevated anxiety and depression at both T2 and T3.

Parental perceptions of their children’s emotional responses also played a role. Parents who reported their children were worried about infection, unsettled by masks, or concerned about their loved ones exhibited higher levels of anxiety and depression. These associations were particularly evident at T2 and, in some cases, persisted through T3.

Children’s limited activities during lockdown—being alone, not doing sports, and spending little time outdoors—were associated with elevated parental symptoms across both timepoints. Notably, parents whose children were unable to see friends during the lockdown also reported higher depressive symptoms at T3 — for example, one parent described their child becoming increasingly withdrawn and lonely after extended isolation, which coincided with the parent’s own reports of persistent sadness and fatigue.

Detailed statistical results for all examined variables are available in Table 2.

### Prediction of anxious and depressive symptomatology by demographic and COVID-related variables

Regression analyses showed that demographic and COVID-related variables significantly predicted anxiety during the lockdown, explaining 63.8% of the variance (*F*(6,112) = 33.85, *p* < 0.001, *R* ^2^ = 0.64). Significant predictors included higher GAD scores at T1, not working less than usual, lack of trust in government measures, and concerns about children being alone, spending little time outdoors, or not engaging in sports.

For depressive symptoms during lockdown, key predictors were higher CESD-R scores at T1, lack of trust in government measures, and children being alone or not engaging in sports. This model explained 41.2% of the variance (*F*(4,112) = 20.59, *p* < 0.001, *R* ^2^ = 0.412).

Post-lockdown anxiety (T3) was best predicted by higher GAD scores at T1, lower parental education, worries about access to primary care, and children being alone, explaining 65.6% of the variance (*F*(4,112) = 54.37, *p* < 0.001, *R* ^2^ = 0.66).

For post-lockdown depression (T3), higher CESD-R and PSS scores at T1, lower parental education, and children being alone were significant predictors (*F*(4,112) = 33.80, *p* < 0.001, *R* ^2^ = 0.54). See Table 3 for full model details.

**Table 3:** Linear regression models for sociodemographic and COVID-related variables associated with anxiety and depression at T2 and T3

**Variable**	**B**	**SE**	**Beta**	***t***	***p***
**Model GAD (T2)**					
GAD (T1)	0.893	0.080	0.661	11.095	< 0.001
I had to work less than usual during the COVID-19 pandemic	−1.972	0.968	−0.117	−2.037	0.044
I consider the measures taken by the government correct and necessary	−0.758	0.345	−0.129	−2.201	0.030
My child was alone during the most recent lockdown	1.216	0.313	0.226	3.886	< 0.001
My child was outdoors during the most recent lockdown	0.859	0.427	0.134	2.011	0.047
My child did sports during the most recent lockdown	−1.323	0.391	−0.226	3.385	0.001
**Model CESD (T2)**					
CESD (T1)	0.768	0.109	0.520	7. 027	< 0.001
I consider the measures taken by the government correct and necessary	−2.641	1.093	−0.180	−2.415	0.017
My child was alone during the most recent lockdown	2.028	0.994	0.151	2.040	0.044
My child did sports during the most recent lockdown	−2.208	1.088	−0.151	−2.029	0.045
**Model GAD (T3)**					
GAD (T1)	0.782	0.070	−0.636	11.212	< 0.001
Education	−0.645	0.236	−0.156	−2.731	0.007
I am worried that primary care is endangered due to COVID-19	1.141	0.325	0.202	3.509	0.001
My child was alone during the most recent lockdown	1.510	0.276	0.308	5.465	< 0.001
**Model CESD (T3)**					
CESD (T1)	0.611	0.094	0.460	6.489	< 0.001
PSS (T1)	0.216	0.096	0.162	2.243	0.027
Education	−3.096	0.674	−0.303	−4.590	< 0.001
My child was alone during the most recent lockdown	3.997	0.794	0.331	5.031	< 0.001

*CESD-R* Center for Epidemiologic Studies Depression Scale – Revised, *GAD-7* Generalized Anxiety Disorder 7, *PSS* Parental Stress Scale, *T1* two weeks before the second nationwide lockdown, *T2* two weeks during the peak of that lockdown, *T3* most recent two weeks following the lockdown

## Discussion

This study found that parents experienced heightened levels of anxiety and depression during the COVID-19 lockdown, which, although reduced, remained elevated in comparison to pre-lockdown levels even after restrictions were lifted. Importantly, the findings highlight that beyond demographic influences, COVID-19-related stressors played a significant role in shaping parents’ mental health outcomes. Key contributing factors included pre-pandemic mental health status, parental education, concerns about disrupted access to primary care, and children being left alone during the lockdown period—both at its height and in the aftermath.

Consistent with previous research [9–13], the present study found that parents of preschool-aged children experienced a significant increase in anxiety and depressive symptoms during the most critical phase of the second nationwide lockdown, compared to pre-pandemic levels with a medium to large effect size. Furthermore, while these elevated symptoms persisted after the lockdown was lifted, their intensity was notably reduced showing a small-to-moderate effect size —an outcome that aligns with earlier findings [15].

In examining the factors most strongly associated with parental mental health, the present study found that demographic variables—particularly changes in work status and parental education—had a significant impact. Parents who experienced a reduction in work hours during the COVID-19 pandemic reported higher levels of anxiety and depressive symptoms during the most critical phase of the nationwide lockdown. This finding aligns with prior research indicating that job-related stressors, such as reduced income [19, 20], income insecurity [7], and job loss [21], negatively affect mental health during crises. However, while bivariate analyses suggested that reduced working hours were linked to higher levels of anxiety and depression, regression analyses controlling for child-related factors during the lockdown revealed a more nuanced picture: working fewer hours was actually associated with lower anxiety symptoms. This pattern may reflect a dual dynamic. On the one hand, parents who experienced job loss or were unable to fulfil their professional responsibilities due to pandemic-related restrictions—often accompanied by fears of dismissal—may have shown elevated anxiety levels. On the other hand, in contexts where job security was maintained, a reduction in workload may have had a protective effect, providing parents with greater opportunities for caregiving and personal recovery.

Parental education also played a significant role, particularly in predicting lower depressive symptoms after the lockdown. The regression models showed that higher educational attainment was one of the strongest demographic predictors of better mental health outcomes at T3. Previous studies have found that higher educational attainment is generally associated with lower levels of psychological distress during the pandemic [17, 21]. This may reflect greater access to coping resources, flexible work arrangements, or higher health literacy. However, during the most acute phases of the pandemic, the severity and universality of the crisis may have temporarily overridden the typical protective effects of higher education, resulting in similarly elevated stress levels across educational backgrounds. Additionally, this effect could be partly explained by the nature of jobs typically held by more educated individuals—positions that often allow for remote work, which may reduce certain stressors while introducing others [21]. It is also important to note that data for earlier time points were collected retrospectively; thus, the stronger correlations observed at T3 may reflect both actual changes over time and potential recall biases.

Notably, the variable that most consistently predicted anxiety and depressive symptoms both before and after the COVID-19 lockdowns was the presence of pre-pandemic mental health symptoms. This finding aligns with previous research demonstrating that a history of mental illness [11] or the presence of chronic conditions [19] significantly increases vulnerability to heightened anxiety and depression during the pandemic. These results underscore the importance of considering pre-existing mental health conditions when assessing the psychological impact of crisis situations such as COVID-19 lockdowns.

In terms of self-related worries, the present study found that a lack of confidence in government measures significantly influenced both anxiety and depression levels in parents during the pandemic. However, this effect diminished once restrictions were lifted. In contrast, parents who expressed concerns about the disruption of primary care services due to COVID-19 exhibited heightened anxiety during the most critical phase of the lockdown, and this anxiety persisted even after the restrictions were eased. This suggests that, while confidence in governmental actions may become less relevant after the peak of the pandemic, concerns regarding the accessibility of primary care continue to play a significant role in sustaining anxiety and depression among parents.

Among all child-related variables, the experience of children being left alone during the lockdown emerged as the most robust and consistent predictor of parental anxiety and depression. It was significantly associated with both outcomes during the lockdown (T2) and remained the strongest predictor at follow-up (T3), even after controlling for pre-pandemic mental health. This highlights the lasting emotional toll that perceived or actual isolation of children may have on parents, particularly when adequate supervision is lacking [43, 44]. Previous research has shown that prolonged isolation can negatively affect children’s emotional well-being, which in turn may heighten parental stress and psychological distress [45]. In contrast, reductions in sports participation contributed to increased distress during the lockdown but did not have a lasting impact. These findings underscore the need to prioritize children's social and emotional well-being during crisis periods, as their experiences can exert a sustained influence on parental mental health.

Our findings should be interpreted in light of certain limitations. First, data from two time points (T1 and T2) were collected retrospectively. Retrospective questions about subjective feelings and mental health are known to be vulnerable to recall bias, particularly because participants tend to recall the past in ways that align with their present state [46]. Consequently, parents'responses may have been influenced by their situation at T3, potentially resulting in an underreporting of challenges faced at T1 and T2. Despite our efforts to make the questions concise and clear, and the fact that the time frame in question was relatively recent to aid memory recall [47], differences between pre- and post-pandemic conditions may have been underestimated. Additionally, the current study did not utilize standardized scales with sufficient methodological validation regarding COVID-related factors. Although the questionnaires were designed to be clear and demonstrated good internal consistency across different time points, the possibility of bias in the responses cannot be completely ruled out. The COVID questionnaire included the item: *"My child was alone during the lockdown."* The responses likely depended on parental interpretation—some parents may have literally left their toddlers alone for a period, while others may have understood the question to mean their child only interacted with immediate family. As a result, drawing definitive conclusions from this item is challenging. Lastly, the study focused solely on parents of preschoolers who had attended preschool prior to the pandemic. Future research is needed to explore the effects of lockdowns on children who were either cared for at home, by non-preschool caregivers or by non-parents.

The present study has several strengths. For the first time, this work examines the mental health post-pandemic of preschool children’s parents in light of the pandemic and examines which factors contribute to the maintaining of anxiety and depressive symptoms. This was performed using a sufficiently powered sample of parents of preschool children, validated measures for a variety of constructs and different timepoint measures. In conclusion, the present study highlights the enduring impact of the COVID-19 pandemic on parental mental health, with anxiety and depression levels remaining elevated in comparison to pre-lockdown levels even after restrictions were lifted. It underscores the importance of considering both pre-existing mental health conditions and COVID-19-related stressors. Notably, among all child-related factors, the experience of children being left alone during lockdown emerged as the most consistent and impactful predictor of sustained parental distress. Additionally, demographic characteristics—particularly parental education—played a significant role, especially after the acute crisis period. These findings suggest that targeted interventions addressing both pre-pandemic mental health vulnerabilities and high-impact situational stressors, such as disruptions to children’s care and routines, to effectively support parents' well-being in future crises.

## Data Availability

The datasets generated and/or analyzed during the current study are available on the Open Science Framework (OSF; DOI: https://doi.org/10.17605/OSF.IO/KVJU5).
